# Sources of ILC2s: diverse locations and precursors

**DOI:** 10.18632/aging.102222

**Published:** 2019-08-30

**Authors:** Xiao-Hong Sun

**Affiliations:** 1 Oklahoma Medical Research Foundation, Oklahoma City, OK 73104, USA

**Keywords:** innate lymphoid cells, thymus, committed T cell precursors, ILC progenitors, ILC2

Group 2 innate lymphoid cells (ILC2s) belong to one of the three ILC subsets (ILC1-3) and can be quickly activated by a variety of stimuli ranging from IL-25, IL-33 and TSLP generated upon epithelial tissue damage to neuropeptides produced by neuronal cells. ILC2s in turn release cytokines, primarily IL-5 and IL-13, to modulate immune functions including recruitment of eosinophils and induction of M2 macrophage differentiation. ILC2s are enriched in mucosal tissues like lung, gut and skin, and thus have ample opportunities to respond to acute insults from viral and parasitic pathogens as well as chronic exposures to allergens and microbiota. Although ILC2s are present in small numbers compared to type2 T helper cells (Th2s), their strategic locations and rapid reactions to diverse stimuli offer them a role as first responders to instruct innate and adaptive immunity.

ILC2 production occurs in three phases: fetal, neonatal and adult. Neonatal ILC2s contribute to the majority of tissue-resident ILC2s as revealed by a lineage tracing study [1]. ILC2s in the lung and visceral adipose tissue are largely stationary and expand locally in response to stimulation. However, ILC2s in the small intestine and skin appear to have a relatively higher rate of turnover, losing the representation of neonatal ILC2s later in life. This may correlate with the rapid renewal of intestinal epithelia and skin, thus requiring replenishment from circulating ILC2s. Alternatively, ILC2s in different tissues use distinct mechanisms for homeostasis. Single cell RNA sequencing data already show that ILC2s in different tissues possess distinct transcriptomes, highlighting the impact of tissue environment on ILC2 differentiation and function [2].

Regardless of the developmental stages at which ILC2s are formed, they are thought to arise from common lymphoid progenitors (CLPs), which are derived from hematopoietic stem cells in fetal liver or bone marrow. In the bone marrow, CLPs progressively lose the potential to differentiate into B lymphocytes and then NK cells to give rise to specialized progenitors capable of generating all three subsets of ILCs [3]. However, newly made ILC2 precursors (ILC2Ps) are more readily detectable than their ILC1 and ILC3 counterparts in the bone marrow. Whether ILC2s found in tissues evolve from ILC2Ps made in the bone marrow or result from de novo differentiation from earlier progenitors is not entirely understood.

Since CLPs or their equivalents also go to the thymus to generate T lymphocytes, it is conceivable that thymic progenitors can also differentiate into ILCs, especially ILC2s, given the rich environment for IL-7 and Notch signals provided by the thymus. Indeed, ILC2s are found in the thymii of wild type mice. However, we and others uncovered the enormous capacity of the thymus to support ILC2 differentiation by blocking the T cell path through impairing the function of E protein transcription factors which are essential for T cell differentiation [4,5]. In E protein deficient mice generated either by expressing the Id1 inhibitor or by gene ablation, 10^5^-10^6^ ILC2s are made in the thymus, and these then populate tissues throughout the body.

Unexpectedly, we have recently found that not only thymic multipotent early T cell progenitors, but also fully-committed T cell precursors can differentiate into ILC2s in vivo and in vitro [6]. Until then, these committed T cell precursors, namely CD4^-^CD8^-^c-kit^-^CD25^+^ thymocytes, had never been found to generate any other cell types besides various subsets of T cells. These findings suggest that ILC2s can originate from diverse precursors in different environments. Although the thymus is primarily designed for making T cells, there are opportunities for ILC2 production. For example, when E protein function is downregulated through transient up-regulation of Id proteins following pre-T cell receptor (pre-TCR) or TCR signaling, some developing T cells could divert to the ILC2 path. It will be interesting to determine if any pathological conditions can augment Id gene expression in the thymus, thus promoting extra ILC2 differentiation. Whether thymus-derived ILC2s possess unique functions remains to be thoroughly investigated. It will also be important to learn whether committed T cell precursors can differentiate into other ILC subsets outside of the thymus, which appears to favor ILC2 differentiation.

**Figure 1:**
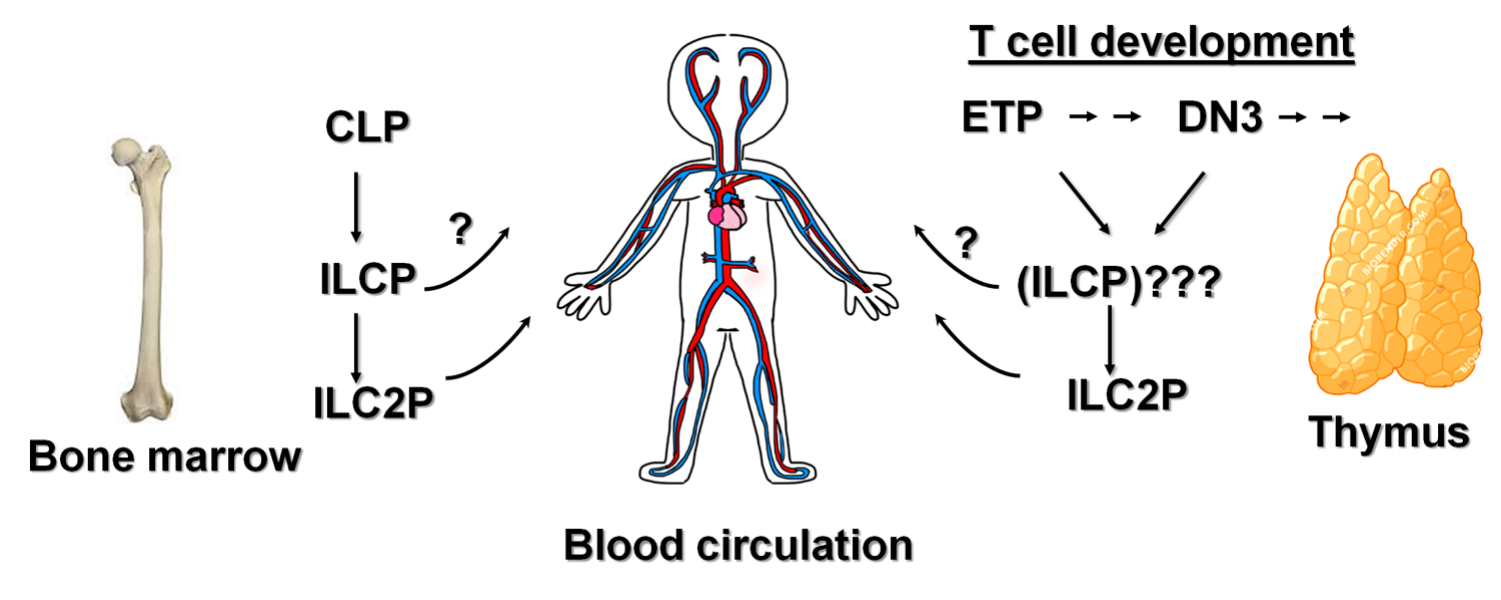
**Contribution of ILC2s by the bone marrow and thymus**. In the bone marrow, a variety of progenitor subsets (collectively called ILCP) have been shown to descend from CLP, and differentiate into all three ILC groups, but immature ILC2s (ILC2P) are more abundant. In the thymus, ETPs which arise from thymus-seeding progenitors generated in the bone marrow, as well as full-committed T cell precursors such as DN3 cells can differentiate into ILC2s, possibly through precursors similar to those found in the bone marrow. Newly made ILC2s and probably their progenitors then circulate in the blood and populate in tissues.

Parabiosis studies showed little exchange of ILC2s between the lungs of two paired wild type mice, thus leading to the notion that ILC2s are mostly tissue-resident [7]. However, when parabionts were created between wild type mice and our Id1 transgenic mice, the latter avidly donated their ILC2s to wild type recipients, causing a 6-fold increase in lung ILC2 counts (Huang, Qian and Sun, unpublished). This result reveals a potential for ILC2s to travel through the blood and populate in different tissues. In humans, ILCs and ILC progenitors are readily detectable in the blood [8] (Figure 1). Increased ILC2 counts have been found in asthmatic patients, consistent with the known function of ILC2s. These observations suggest that the production of ILCs, at least in humans, is more dynamic than implied by data from mouse studies. These human ILCs or ILC progenitors are presumed to originate in the bone marrow but the capacity of the thymus to contribute to the pools has not been investigated. Considering that the thymus undergoes involution with age, its output of ILCs or ILC progenitors could vary over time. A young thymus may be more active but an old thymus may retain the ILC producing potential while being impaired in T lymphopoiesis Understanding the thymic contribution to ILC homeostasis may help explain the age-related immunological diseases such as the higher prevalence of asthma in children.
